# A Graphene Oxide–Thioamide Polymer Hybrid for High‐Performance Supercapacitor Electrodes

**DOI:** 10.1002/smsc.202300013

**Published:** 2023-04-05

**Authors:** Włodzimierz Czepa, Samanta Witomska, Paolo Samorì, Artur Ciesielski

**Affiliations:** ^1^ Faculty of Chemistry Adam Mickiewicz University Uniwersytetu Poznańskiego 8 61614 Poznań Poland; ^2^ Center for Advanced Technologies Adam Mickiewicz University Uniwersytetu Poznańskiego 10 61614 Poznań Poland; ^3^ Nanochemistry Laboratory Institut de Science et d'Ingénierie Supramoléculaires (I.S.I.S.) Université de Strasbourg, CNRS, ISIS 8 alleé Gaspard Monge 67000 Strasbourg France

**Keywords:** energy storage, graphene oxide, supercapacitors, thioamide polymer

## Abstract

The controlled chemical functionalization of graphene oxide (GO) represents a powerful strategy to finely tune its physical and chemical properties toward applications in energy storage. Herein, an unprecedented approach for the GO modification with thioamide‐based polymers featuring numerous heteroatoms (S,N,O) is reported, which is instrumental for achieving superior electrochemical performance in symmetric supercapacitors. While the electrochemical investigations in aqueous electrolytes reveal specific capacitance of 221 F g^−1^ at 1 A g^−1^, the use of organic media allows the specific capacitance to be boosted up to 340 F g^−1^. Additionally, the increase of operating window yields energy densities as high as 94.4 Wh kg^−1^, thereby exceeding state‐of‐the‐art performances of GO‐based supercapacitors. Furthermore, the symmetric devices exhibit great robustness in both aqueous and organic electrolytes as evidenced by an excellent stability after 5000 working cycles (>98% in H_2_SO_4_ and >90% in TEABF_4_/ACN).

## Graphical abstract

The functionalization of graphene oxide with thioamide‐based polymer, to boost electrochemical performance of the material as a result of introduction of heteroatoms (N,S) into the structure providing redox active centers, is presented. The symmetric supercapacitor device is investigated in aqueous and organic electrolyte, reaching high energy denisty of 94.4 Wh kg^−1^ facilitated by wide potential window.

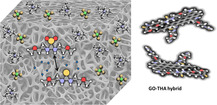

## 1 Introduction

The ever‐growing energy needs call for the development of new power sources and sustainable energy storage systems. Supercapacitors (SCs) represent an attractive portable energy storage alternative to state‐of‐the‐art devices, e.g., batteries, as they combine very high cycling stability, ultrafast charging, high range of operating temperatures and improved durability, and most importantly high power density.^[^ ^1^, ^2^, ^3^ ^]^ However, several obstacles need to be overcome to render SCs ready for being used in daily applications, e.g., to power portable electronic devices. The existing major problems to be solved include: 1) the lack of industrial standardization such as general specifications, electrodes, and electrolyte specifications; 2) low rated voltages, which require preparation of stack of connected SC components for practical applications; 3) relative low energy densities, which result in the need of constructing bulky, noncompact, and relatively heavy devices.^[^ ^4^, ^5^ ^]^ Various types of materials have been investigated for application in energy storage devices exploiting either their electrochemical double‐layer (ECDL) formation or pseudocapacitance (PS) provided by Faradic reactions taking place on redox active sides on electrode–electrolyte interface.^[^ ^6^, ^7^, ^8^, ^9^ ^]^ In this context, different forms of carbon‐based materials including activated carbon, carbon nanotubes, and graphene‐based materials have been studied in the last decade.^[^ ^10^, ^11^, ^12^ ^]^ To enhance their electrochemical characteristics, these materials have been modified by increasing their porosity, which rules the electrolyte penetration within the electrode material and therefore it allows control over the kinetics of charge/discharge processes. Moreover, to provide additional pseudocapacitative behaviors, heteroatoms such as N, P, S, and O have been introduced.^[^ ^13^, ^14^, ^15^ ^]^ During the last few years, numerous studies have been reported on the exploration of 2D materials toward enhanced efficiency in energy storage density, mainly achieved by increasing their specific surface areas, taking advantage of their high electrical conductivity, and by developing synthetic protocols that can be potentially upscaled.^[^ ^16^, ^17^ ^]^

Among 2D materials, graphene oxide (GO) can be produced in large scale at moderate costs, and it is known to exhibit unique physical and chemical properties including high mechanical strengths and ease of processability. Furthermore, the presence of oxygen containing groups (carboxyls, alcohols, and epoxides) on the surface and rim of GO's sheets enables the use of different synthetic pathways for its covalent and noncovalent modification.^[^ ^18^, ^19^, ^20^, ^21^, ^22^, ^23^ ^]^ The chemical functionalization of GO was amply demonstrated to be a powerful strategy to tune its properties toward desired application in (electro)chemical sensing, energy storage, water and gas purification as well as biomedical technologies.^[^ ^24^, ^25^, ^26^, ^27^, ^28^ ^]^ However, GO exhibits poor electrical characteristics when compared to pristine graphene due to the presence of multiple structural defects and the aforementioned oxygen‐containing groups acting as traps and scattering sites for efficient charge transport. Moreover, the GO itself is highly hydrophilic and it can be easily penetrated by solvents molecules causing swelling of the material.^[^ ^29^ ^]^ The phenomena significantly influence the structure and result in worsening of long‐cycle performance of the electrode causing low stability.^[^ ^30^ ^]^ Nonetheless, various chemical and physical methods for the GO reduction can be used to restore the conjugation between carbon atoms within GO yielding reduced graphene oxide (rGO). The latter is electrically conducting and it can be used both as electrode or semiconductor for the fabrication of optoelectronics devices.^[^ ^31^ ^]^ Interestingly, the reduction and functionalization of GO can be accomplished simultaneously when molecules bearing primary amines are employed to covalently modify GO via condensation between the oxygen functional groups of GO (mainly epoxides) and NH_2_ units of ad hoc molecules. This results in the dramatic decrease of oxygen content and restoration of GO conductivity.^[^ ^32^, ^33^ ^]^ Noteworthy, a moderate amount of oxygen is also desired in carbon‐based materials when employed as SC's electrode material, as they provide pseudocapacitative characteristics.^[^ ^34^, ^35^ ^]^ Previous works have been focussed on the influence of oxygen content on SC performance in GO/rGO‐based electrodes. Harsh reduction conditions might cause drastic decrease in specific capacitance due to lowered pseudocapacity effect.^[^ ^36^ ^]^ The today's performance of most GO/polymer hybrid materials employed as electrodes in SCs, as quantified by its specific capacitance, amounts typically in the range of 100–300 F g^−1^.^[^ ^37^, ^38^, ^39^ ^]^ Importantly, the crucial obstacle hampering the practical application of GO‐based hybrids in SCs is the low energy density, being usually two orders of magnitudes lower than in case of Li‐ion batteries and typically reaching values up to 5–15 Wh kg^−1^ in case of aqueous electrolytes.^[^ ^40^ ^]^ Notably, Song and co‐workers showed that the use of 1‐butyl‐3‐methyl‐imidazolium tetrafluoroborate electrolyte allows a high energy density of 51 Wh kg^−1^ to be obtained, demostrating the high potential of organic electrolytes for widening of the potential window.^[^ ^41^ ^]^ Most of reported examples of GO‐based SCs comprise aqueous electrolytes, which are relatively cheap, easily available, and provide good ion conductivity. However, the operating voltage windows of such SCs are limited by the thermodynamic water decomposition at 1.23 V.^[^ ^42^ ^]^ According to theoretical predictions, the use of higher voltage windows, which might be modulated by the choice of the electrolyte, represents appealing strategy toward increased energy density and power density performance of SCs.^[^ ^43^, ^44^ ^]^ In particular, organic electrolytes, including ionic liquids, represent attractive solutions when employed in carbonaceous‐based SCs.^[^ ^45^, ^46^ ^]^ Among them, tetraethylammonium tetrafluoroborate (TEABF_4_) exhibits very high conductivity (10.55 mS cm^−1^) and very high oxidation and reduction limiting potentials defining an operative voltage window in the range of −3.0 to 3.6 V.^[^ ^47^ ^]^ Hence, organic electrolytes are particularly suitable for the development of hybrid SCs with excellent performance by mastering both ECDL and PS mechanisms of energy storage.^[^ ^48^ ^]^ To generate new electrode materials mastering both mechanisms, we exploited a supramolecular approach comprising the synthesis of a new thioamide‐based polymer (THA) and its grafting to GO yielding GO‐THA porous hybrids. The latter have been used as electrode material in symmetric SC devices, displaying gravimetric capacitance as high as 221 and 340 F g^−1^ at 1 A g^−1^ in 1 M aqueous H_2_SO_4_ and 1M TEABF_4_ in ACN, respectively. Moreover, the enlarged operating potential window allowed the achievement of energy density of 94.4 Wh kg^−1^, being several times greater than typical values for carbonaceous SCs (Table S6, Supporting Information).

## 2 Results and Discussions

### 2.1 Synthesis

**Figure 1:**
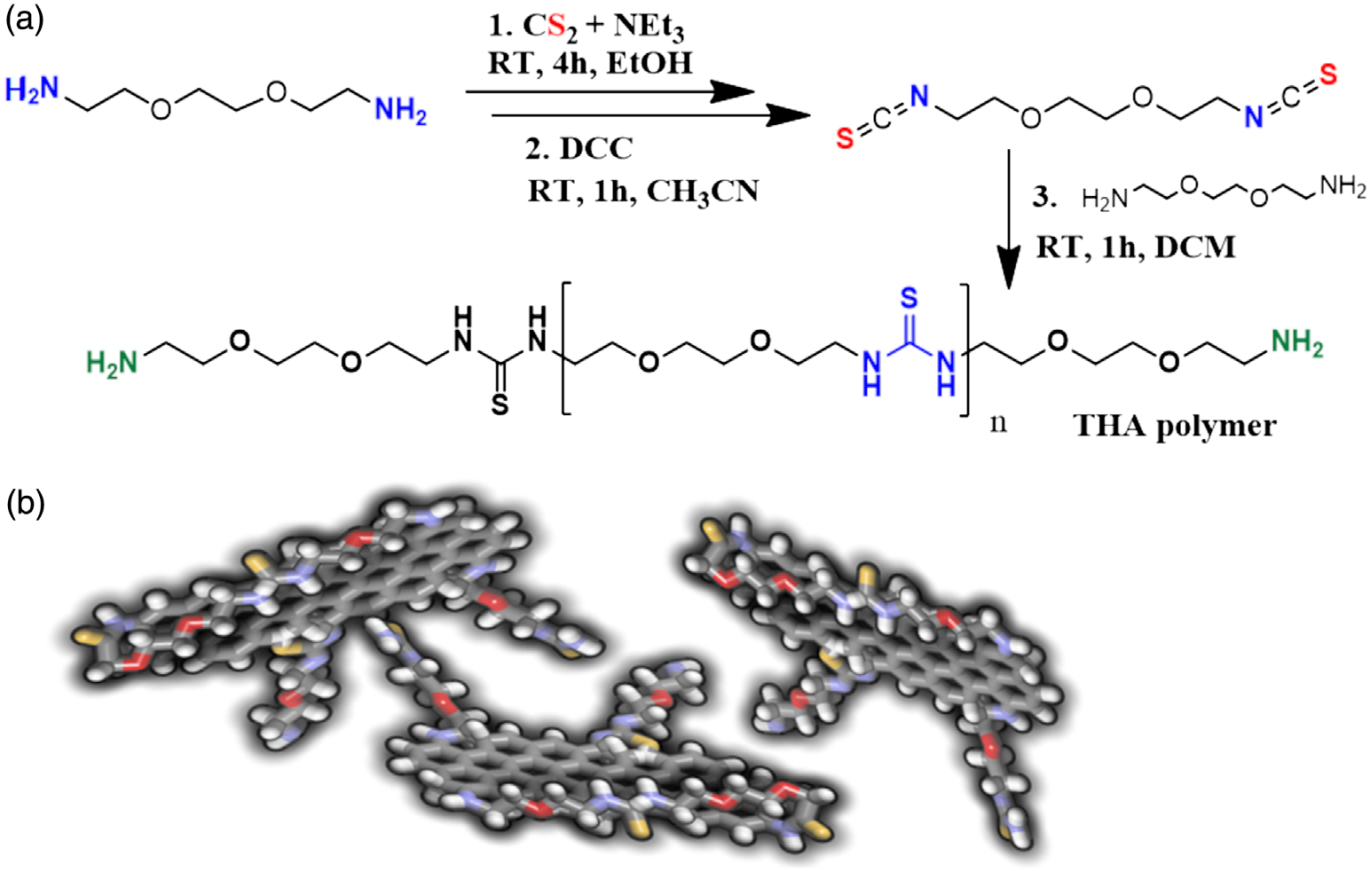
a) Scheme of the synthesis of the THA polymer. b) Model of the GO‐THA composite.

The thioamide polymer (THA) was synthesized in a three‐step reaction. First, modified Hofmann rearrangement was employed to substitute terminal primary amines of 2,2′‐(ethylenedioxy)bis(ethylamine) with dithiocarbamide salt by reacting the former with carbon disulfide in basic conditions. Subsequently, dithiocarbamide salt was transformed into diisothiocyanate.^[^ ^48^ ^]^ Finally, the condensation of diisothiocyanate and 2,2′‐(ethylenedioxy)bis(ethylamine) yielded the thioamide (THA) polymer. The condensation reaction time was set to 1 h to avoid complete polymerization and therefore insoluble polymer precipitation (**Figure** **1**). The as‐prepared THA was used to modify GO through the ring opening reaction of GO's epoxide‐driven nucleophilic attack of THA's terminal primary amines, as well as the esterification of carboxyl moieties located mainly on the GO sheet edges. The former reaction is accomplished thanks to the tautomerization of active sulfides (thioamide grouping).^[^ ^49^ ^]^ The ratio GO:THA 1:10 (w/w) was applied to maximize the functionalization degree of GO and to avoid formation of nonhomogeneous product.

### 2.2 Characterization

**Figure 2:**
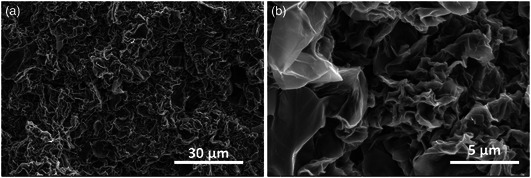
SEM images of GO‐THA hybrid.

^1^ H and ^13^C NMR analysis was carried out to attain qualitative information on the chemical structure of THA (Figure S1, Supporting Information). ^1^H spectra revealed the presence of signals at *δ* 7.54 (s, 2 H), 3.68–3.44 (*m*, 12 H), and 2.67 (*t*, NH_2_) which are assigned to secondary amine, ethylene units, and terminal amines hydrogens of THA, respectively. The average molecular weight of the polymer size was estimated as 1958 g mol^−1^ by MALDI‐TOF with MS detector (Figure S2, Supporting Information). Scanning electron microscopy (SEM) and transmission electron microscopy (TEM) studies of the GO‐THA hybrid material revealed a dense, homogeneous, wrinkled, porous morphology (**Figure** **2** and S3, Supporting Information). Such porosity is especially beneficial for the subsequent electrochemical experiments, as it is one of the factors enabling efficient electrolyte ion transportation. Moreover, the energy‐dispersive X‐Ray (EDX) mapping (Figure S4, Supporting Information) showed an even distribution of the heteroatoms (S, N) introduced upon functionalization with the THA polymer.

**Figure 3:**
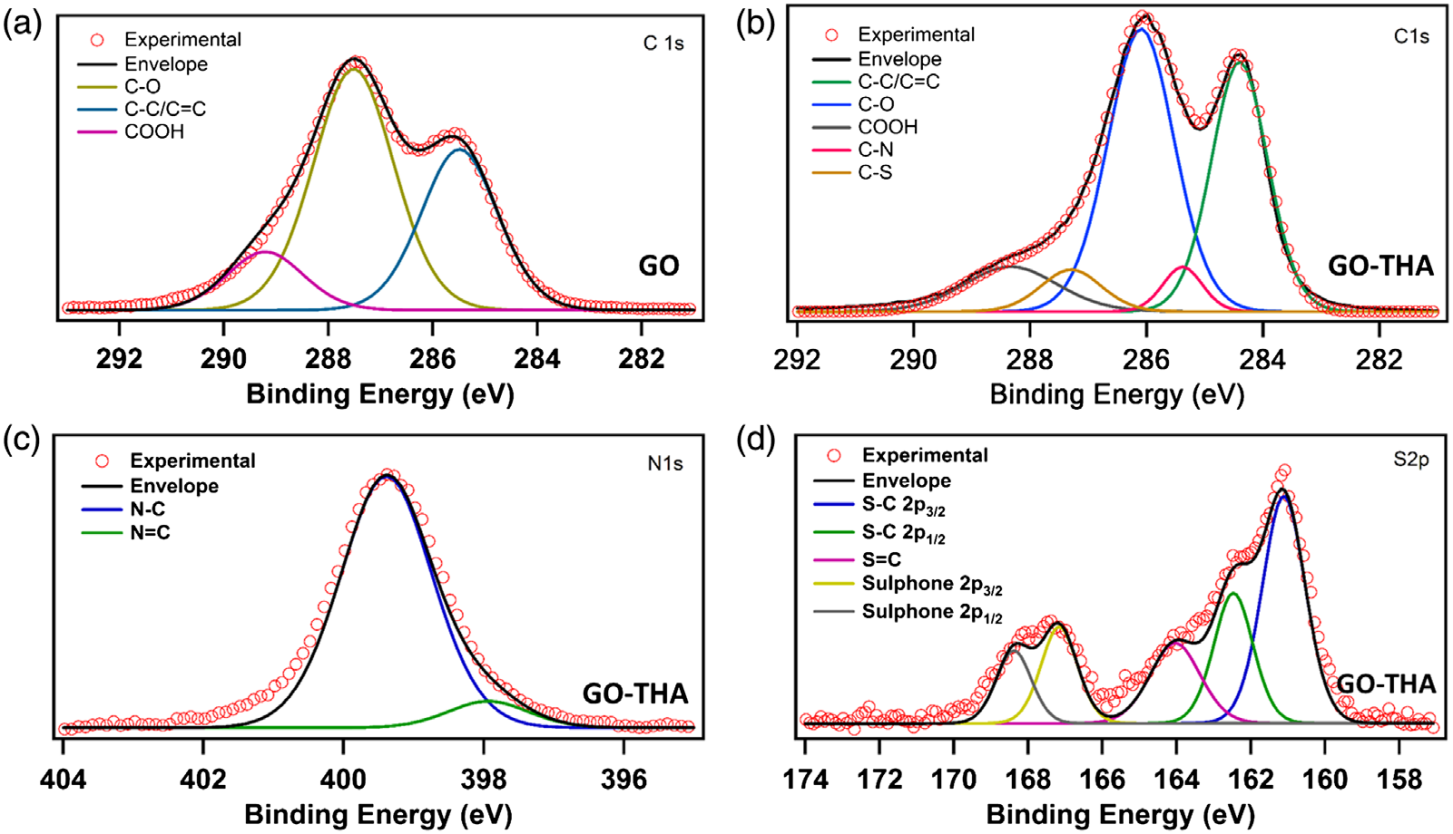
High‐resolution XPS spectra of GO: a) C 1s, and GO‐THA: b) C 1s, c) N 1s, and d) S 2p.

X‐ray photoelectron spectroscopy (XPS) measurements of pristine GO, THA, and GO‐THA hybrid were performed to cast further light onto the chemical composition of the materials. The survey spectra confirm the presence of introduced heteroatoms (N, S) into the GO structure (Figure S5, Supporting Information) High‐resolution C 1s spectrum of GO (**Figure** **3**a) exhibited typical peaks at 284.6, 286.4, 287.5, and 288.3 eV corresponding to the C–C, C–O, C=O, and COOH groups, respectively. N 1s and S 2p spectra of THA displayed characteristic peaks confirming its structure along with the emergence of resonance effect according to S 2p high‐resolution spectra featuring C=S and C–SH peaks at 161.5 and 163 eV, respectively. (Figure S6, Supporting Information). The C 1s spectra of pristine GO and GO‐THA hybrid evidenced major differences. In particular, in the latter two new peaks appeared: the C–N bond (287.3 eV) arising from the covalent attachment of terminal primary amines of THA to GO's epoxides, and the band at 285.5 eV which is assigned to C–S bond and whose presence has confirmed the formation of thioamide.^[^ ^50^, ^51^ ^]^ On the other hand, the N 1s spectra of GO‐THA provided valuable insight into the composition of obtained material. While the major peak at 399.5 eV (Figure 3c) can be ascribed to N–C bond of thioamide and secondary amine grouping, the existence of a peak at 398.1 eV is indicative of a resonance effect taking place in the thioamide moiety [R1–NH–C(=S)–NH–R2]↔[R1–HN–C(SH)=N–R2].^[^ ^52^ ^]^ The S 2p spectra of GO‐THA revealed characteristic sulfur–carbon bonds (at 161.0, 162.1, and 164 eV). Moreover, partial oxidation of sulfides to sulfones was monitored (167.0 and 168.5 eV), occurring simultaneously while functionalizing the GO. This phenomenon is beneficial especially in view of electrochemical properties of GO‐THA, as redox reversible reactions allow the introduction of an additional PS effect and improved the SCs’ performance. Notably, the chemical composition of GO‐THA hybrid displayed significant degree of functionalization as revealed by a nitrogen and sulfur content amounting to 9.23% and 3.93%, respectively, according to XPS data. Such values are in good agreement with elemental analysis (Table S1, Supporting Information).

Fourier transform infrared spectroscopy (FTIR) spectra of GO and GO‐THA are shown in Figure S7, Supporting Information. The GO spectrum displayed the well‐known bands at 1731 cm^−1^ (C=O stretching), 1620 cm^−1^ (C=C), and stretching of epoxy moieties C–O–C at 1047 cm^−1^. Moreover, one can observe the broadband between 3000 and 3500 cm^−1^, due to the stretching vibration of the hydroxyl groups. As a result of the functionalization, a strong peak appeared at 1568 cm^−1^ that can be ascribed to C=N–H stretching vibrations which is characteristic for thiourea resonance structure. Moreover, one can observe typical –CH_2_–symmetric and asymmetric stretching at 2864 and 2923 cm^−1^, respectively, arising from ethylene grouping present in the polymer.

The functionalization of GO with THA yielded a threefold increase in the specific surface area of 371 m^2^ g^−1^ according to Brunauer–Emmett–Teller (BET) N_2_ adsorption–desorption investigations (Table S2, Supporting Information). The hysteresis of type H3 (Figure S8, Supporting Information) indicates the slit‐like mesopores being present in the material, which is in correlation with an average pore diameter of 3.1 nm. Powder X‐ray diffraction (XRD) investigations were performed to study the crystallinity of the materials. Wide‐angle X‐ray scattering (WAXS) of the pristine GO displayed only the (002) reflection of stacked GO sheets which appears as a typical sharp peak at ≈10.01° (see Figure S9, Supporting Information), indicating an interlayer spacing of 0.87 nm, in accordance with previously reported values.^[^ ^53^, ^54^ ^]^ Conversely, the THA polymer exhibited two broad peaks at ≈22° and a secondary peak at ≈8.3°. Interestingly, GO‐THA hybrid featured a peak at ≈8.15°, suggesting an increased interlayer spacing between adjacent GO sheets up to 1.05 nm. Moreover, due to functionalization a new broad peak at 23.4° originating from an amorphous THA phase can be identified. Raman spectroscopy provided useful information on the quality and degree of functionalization of GO within GO‐THA hybrid. The relative ratio between D and G peaks of GO and GO‐THA (Figure S10, Supporting Information), which are located around 1350 and 1600 cm^−1^ respectively, confirms the highly efficient functionalization. In particular, the increase in the *I*_D_/*I*_G_ ratio from 0.87 to 1.14 in GO‐THA reflects the enhancement in structural and electronic disorder after grafting THA polymer, which is due to the enhancement in the *sp* ^3^ carbon atoms content in the hybrid, in line with previous reports on GO functionalization.^[^ ^55^, ^56^ ^]^ Thermogravimetric analysis was performed to investigate thermal stability of GO, THA polymer, and GO‐THA. The GO curve displayed ≈45% weight loss in the range of 150–300 °C due to the degradation of oxygen functional groups. THA exhibited an average thermal stability up to 210 °C, typical for organic compounds, with a rapid decomposition and weight loss of 60%. The GO‐THA curve revealed a slight mass decrease over 100 °C associated with surface water removal, along with a slow degradation over 150 °C eventually up to 48% mass loss. Overall, based on the thermal gravimetric analysis (TGA) analysis the relative mass composition of GO‐THA is estimated as 80:20 GO:THA (Figure S11, Supporting Information).

### 2.3 Electrochemical Studies

**Figure 4:**
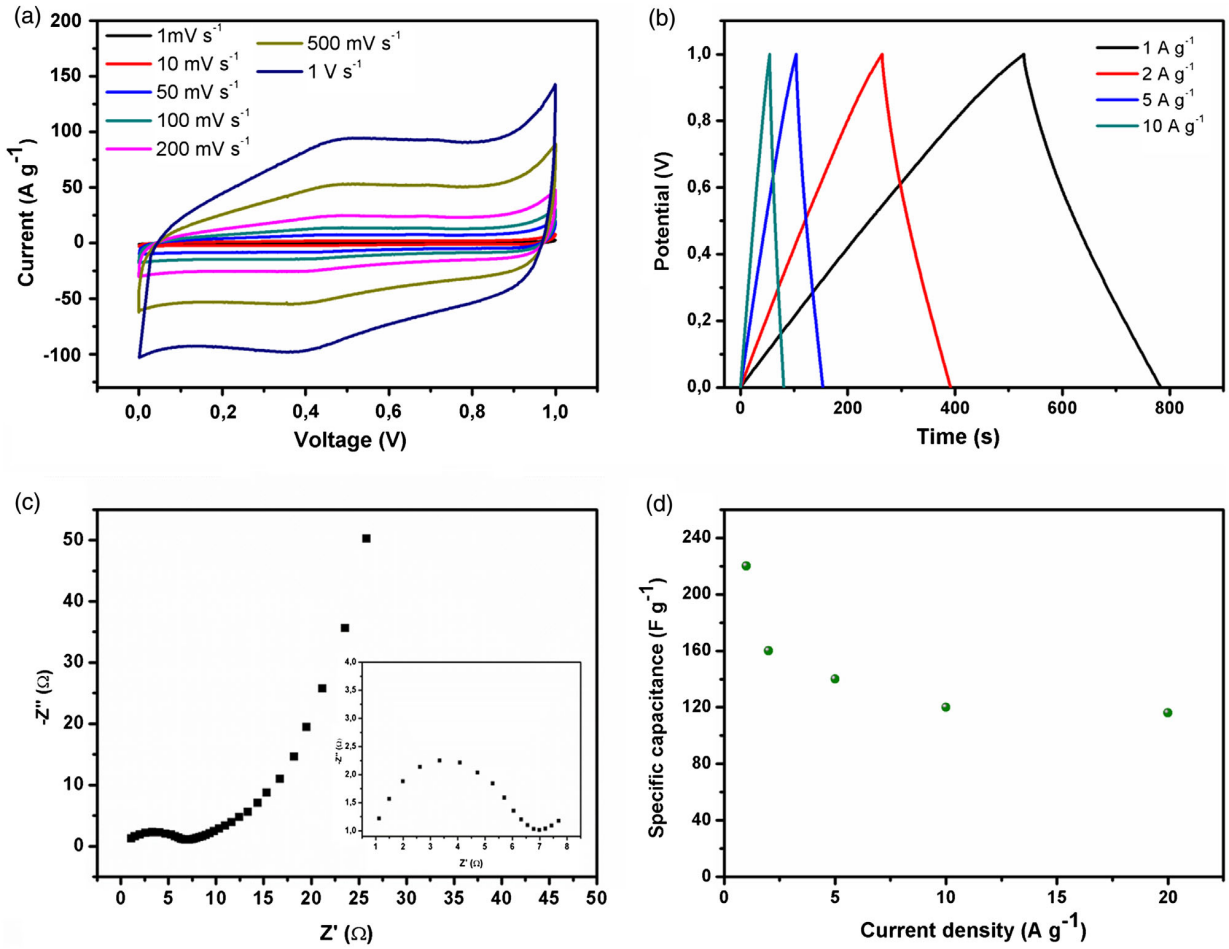
Electrochemical characterizations of symmetric SC in 1 m aqueous H_2_SO_4_: a) CV, b) GCD curves, c) electrochemical impedance spectroscopy, and d) specific capacitance at different current densities.

**Figure 5:**
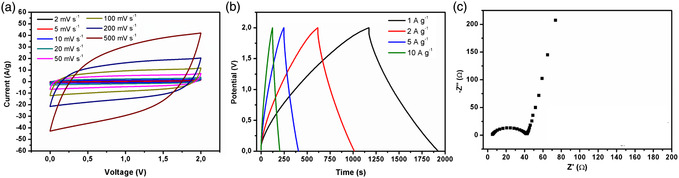
Electrochemical characteristics of GO‐THA electrodes in 1 m TEABF_4_ in ACN: a) CV, b) GCD curves, and c) electrochemical impedance spectroscopy and proposed electrical circuit.

The electrochemical performance of the developed material when integrated in 2‐electrodes SC devices was investigated in solid state symmetric setup embedded in CR2030 coin cell. The use of aqueous electrolyte (1M H_2_SO_4_) provided excellent wettability mainly due to presence of numerous electronegative atoms (O, N, S) in GO‐THA hybrid. The electrolyte–material interface displayed a good conductivity, reaching values of 140 S cm^−1^ (Figure S12a, Supporting Information). The most important electrochemical characteristic is portrayed in **Figure** **4**: the displayed cyclic voltammetry (CV) profile exhibits very good electrode performance. The quasirectangular shape of CV, characterized by small peaks at around 0.4 and 0.5 V, provides evidence for the slight coparticipation of a redox reaction in the energy storage mechanism. The nearly triangular galvanostatic charge–discharge (GCD) curves indicate excellent reversibility and effective charging and discharging of the electrodes. Notably, the electrode does not show any IR drop, which might indicate internal resistance, confirming a negligible charge loss due to resistance effect. Such feature is also confirmed by Nyquist plot revealing solution resistance (*R*_S_) and charge resistance transfer (*R*_CT_) factors of 0.9 and 6.0 Ω, respectively (Figure 4c). Interestingly, the GO‐THA‐based device exhibited high specific capacitance of 221 F g^−1^ at current density of 1 A g^−1^ combined with a great capacitance retention while increased current density up to 20 A g^−1^ (120 F g^−1^). Moreover, the stability tests confirmed outstanding performance even after 5000 cycles, with a decrease in the initial capacitance limited to ≈2% (Figure S12b,c, Supporting Information). In general, the operating potential window determines the performance of energy storage devices (Equation (2)). It has been shown that its widening allows both energy density and power density to be boosted. Toward this end, we have employed 1 m TEABF_4_ in acetonitrile as the electrolyte which made it possible to increase the potential window up to 2 V. The CV profiles in **Figure** **5**a show excellent performance, especially in the range of 2–500 mV s^−1^, as the last curve (500 mV s^−1^). Notably, the electrode exhibits great pseudocapatitative behavior, which can be only observed for low scan rates value (Figure S13, Supporting Information), as the Faradic peaks flatten with subsequent scan rates increment.

The specific capacitance of device calculated from GCD exceeds the value obtained in aqueous media and reaches 371 F g^−1^ at current density of 1 A g^−1^, standing out among other GO‐related materials (Figure 5b). The electrode also exhibits great capacitance retention at higher current densities reaching 250 F g^−1^ at 20 A g^−1^(Figure S14, Supporting Information). Significantly, the increase in the operating window allowed notable increase of the energy density up to values as high as 94.4 Wh kg^−1^ with the power density reaching 333 mWh cm^−3^, being exceptional performance for energy storage applications. Moreover, the GO‐THA electrodes exhibited high areal and volumetric capacitance of 556.5 mF cm^−2^ and 85.6 F cm^−3^, respectively (Table S3, Supporting Information). According to Nyquist plot, *R*_S_ and *R*_CT_ factors can be evaluated by fitting the electrochemical impedance spectroscopy (EIS) spectra on the equivalent circuit proposed on Figure S15, Supporting Information. The charge transfer resistance reached values as low as 32 Ω, thereby providing excellent kinetics of electrochemical processes (Figure 5c). Notably, the attained conductivities of the electrode material correspond to 48 S cm^−1^ (Figure S15a, Supporting Information).

## 3 Conclusions

We have demonstrated the covalent functionalization of GO with thioamide‐based polymer, as an efficient route to decorate in a robust fashion the surface of GO with different heteroatoms. The multiscale characterization of GO‐THA by means of XPS, SEM‐EDX, Raman, XRD, and FTIR analysis revealed significant amount of polymer present in the hybrid with high nitrogen and sulfur content. The GO‐THA hybrid was employed as electrode material in symmetric SCs that were investigated in both aqueous (1 m H_2_SO_4_) and organic electrolyte (1 m TEABF_4_ in ACN). In both cases the electrodes exhibited excellent conductivity and very good specific capacitance amounting to 221 and 340 F g^−1^, respectively. Notably, the use of organic electrolyte made it possible to widen the potential window resulting in very high volumetric capacitances of 85.6 F cm^−3^ and energy density of 94.4 Wh kg^−1^, being several times higher than typical energy densities of other carbonaceous materials.

## 4 Experimental Section

4.1

4.1.1

##### Materials

All the compounds and components required for the synthesis including triethylamine, carbon disulfide, 2,2′‐(ethylenedioxy)bis(ethylamine), dicyclohexylcarbodiimide, electrolyte components tetraethylammonium tetrafluoroborate, acetonitrile (electronic grade), separators Whatman glass microfiber filters, binder poly(tetrafluoroethylene), and 1‐methyl‐2‐pyrrolidinone were purchased from Sigma–Aldrich. Conductive carbon black Super P (H30253) was acquired from Alfa Aesar and carbon AvCarb P75 substrate was obtained from FuelCellStore. The GO was acquired from Graphenea (4 mg mL^−1^ solution).

##### Synthesis of Polymer THA

A 4 mL of ethanol was poured into a round bottom glass placed in ice cooled bath and subsequently 3.9 mL of triethylamine (4 eq, 28 mmol) and 4.2 mL of carbon disulfide (10 eq, 70 mmol) were added. Then, 1.04 g of 2,2′‐(ethylenedioxy)bis(ethylamine) dissolved in 2 mL of ethanol was droplet added while the reaction mixture was intensively stirred. After 5 min the round bottom glass was taken out from the ice cooled bath and stored at room temperature for 4 h. Following, a pale‐yellow precipitate was filtrated and rinsed with cooled ethanol to yield the final product (yield = 97%). To the solution of 0.496 × g *N*,*N*′‐dicyclohexylcarbodiimide (2 eq, 2.4 mmol) in acetonitrile, 0.68 g of triethylammonium ((ethane‐1,2diylbis(oxy))bis(ethane‐ 2,1‐diyl))dicarbamodithiolate (1 eq, 1.2 mmol) dissolved in 5 mL of acetonitrile was added dropwise. The reaction was carried out for 1 h at RT. Subsequently, the white precipitate was filtered, and 10 mL of water was added to the yellow solution, followed by three times extraction with DCM. The combined organic fractions were washed with brine, dried over anhydrous Na_2_SO_4_, and evaporated till fully exsiccated. The crude product was dissolved in a small volume of cold ethyl acetate and placed in the ice bath for 1 h to enable the precipitation of the residual dicyclohexylurea (DCU). After filtration, the solution was evaporated under the vacuum to give the isothiocyanate product as a yellow oil (60% yield). 0.68 × g of 1,2‐bis(2‐isothiocyanatoethoxy)ethane (1 eq, 2.9 mmol) was dissolved in 10 mL of DCM and added dropwise 0.42 mL of 2,2′‐(ethylenedioxy)bis(ethylamine) (1 eq, 2.8 mmol) mixed with constant stirring in RT. After 2 h, additional amount of 2,2′‐(ethylenedioxy)bis(ethylamine) (0.1 mL) was added and stirred for 15 min. The precipitate was filtered and dried to yield product (30% yield).

##### Preparation of GO‐THA Hybrid Material

A mixture of DMF (10 mL) and aqueous GO (10 mL, 4 mg mL^−1^) was sonicated for 15 min and then 10 mL of THA polymer in DMF (40 mg mL^−1^) was added. The mixture was rigorously stirred overnight and heated at 90 °C. The resulting powder was then filtered and washed several times with DMF and copious amount of water to remove the unreacted THA polymer. The product was then freeze‐dried for 24 h under vacuum to obtain black solid foam‐like material.

##### Characterization Techniques

FTIR spectra were recorded in the mid‐IR range (400–4000 cm^−1^) by using a Perkin Elmer Spectrometer (Spectrum Two) equipped with ATR Diamond. XPS analyses were carried out on a Thermo Scientific KAlpha X‐ray photoelectron spectrometer with a basic chamber pressure of ≈10–9 mbar and an Al anode as the X‐ray source (X‐ray radiation of 1486 eV). X‐ray powder diffraction (XRD) experiments were conducted on powder specimens using Bruker ASX D8 Advanced equipped with Cu anode with K*α* radiation (*λ* = 1.5418 Å). Diffraction patterns were collected at room temperature in the scattered angular range between 6° and 40° with an angular resolution of 0.02° per step and a typical counting time 4 of 10 s per step. The specific surface area was measured by using a Micromeritics ASAP 2050 surface area and porosity analyzer. Prior to the BET measurements, the samples were outgassed for 12 h at 100 °C. Adsorption isotherms were calculated for nitrogen adsorption at 77 K and pressures up to 1 bar. SEM characterization was carried out by means of a FEI Quanta 250 FEG instrument with EDX analyses. High‐resolution transmission electron microscopy (HR‐TEM) was performed with a Hitachi HT7700 transmission electron microscope. TGA decomposition curves are recorded in the range 25–700 °C operating under nitrogen atmosphere, with a thermal step of 10 °C min^−1^ on a Mettler Toledo TGA/SDTA851e system. Raman spectra were recorded by a Renishaw microscope with a 100× objective, laser excitation wavelength of 532 nm, and laser power of 0.05%. The silicon peak at 520.3 cm^−1^ was taken as reference for wavenumber calibration. Point of zero charge of adsorbent was determined following previously reported method. Mass spectra were collected on MALDI‐TOF mass spectra Ultrafle Xtreme Bruker.

##### Preparation of SCs

The working electrodes were prepared by mixing of GO‐THA hybrid (80 wt%, 8 mg), carbon black (10 wt%, 1 mg), and poly(tetrafluoroethylene) (PTFE) (10 wt%, 1 mg) in a probe with 1 mL of *N*‐methylpyrrolidone (NMP) and sonicated for 15 min to achieve homogeneous suspension. Consequently, the material was deposited on carbon substrate providing 2 mg of active material per electrode (AvCarb‐P75) and dried under vacuum (80 °C). The electrodes were assembled in CR2032 coin cell using Whatman glass microfiber filters as a separator and several drops of the given electrolyte, i.e., 1 m aqueous H_2_SO_4_ or 1 m tetraethylammonium tetrafluoroborate in acetonitrile.

##### Electrochemical Measurements

The CV study was performed in the range of 0‐2 V with scan rates spanning from 2 to 1000 mV s^−1^. GCD curves were recorded from 0–2 V at different current densities (1–20 A g^−1^) and used to calculate specific capacitance using following formula^[^ ^49^ ^]^

(1) $$E=\frac{2\times I\times \Delta t}{m\times \Delta V}$$

where *I* is a current density, Δ*t* is the discharge time, *m* is a mass of the electrode, and Δ*V* is a potential window. EIS and conductivity of electrodes were taken in the frequency range from 200 kHz to 1 mHz with an amplitude of 10 mV. The energy density (Equation (2)) and power density (Equation (3)) were calculated as given

(2) $$E=\frac{C\times \Delta V^{2}}{3.6\times 2}$$

(3) $$P=\frac{E\prime }{\Delta t}\times 3600$$

where *E* is the energy density (Wh kg^−1^), *C* is the gravimetric capacitance, Δ*V* is the discharge voltage range, *P* is the power density (in mWh cm^−3^), *E*′ is the volumetric energy density, and Δ*t* is the discharge time. CVs, GCD curves, and EIS were recorded using EC‐LAB VMP3 (BioLogic Science Instrument).

## Conflict of Interest

The authors declare no conflict of interest.

## Supporting information

Supplementary Material

## Data Availability

The data that support the findings of this study are available from the corresponding author upon reasonable request.
