# Supplementary material for: A Graphene Oxide–Thioamide Polymer Hybrid for High‐Performance Supercapacitor Electrodes
Source: Small Sci. 2023 Apr 5;3(5):2300013. doi: 10.1002/smsc.202300013 (PMC11935813; doi:10.1002/smsc.202300013)
Supplement: Supplementary file 1 — Supplementary Material [file SMSC-3-2300013-s001.pdf]

Supporting Information

A graphene oxide – thioamide polymer hybrid for high performance supercapacitor electrode

Włodzimierz Czepa, Samanta Witomska, Paolo Samorì and Artur Ciesielski**

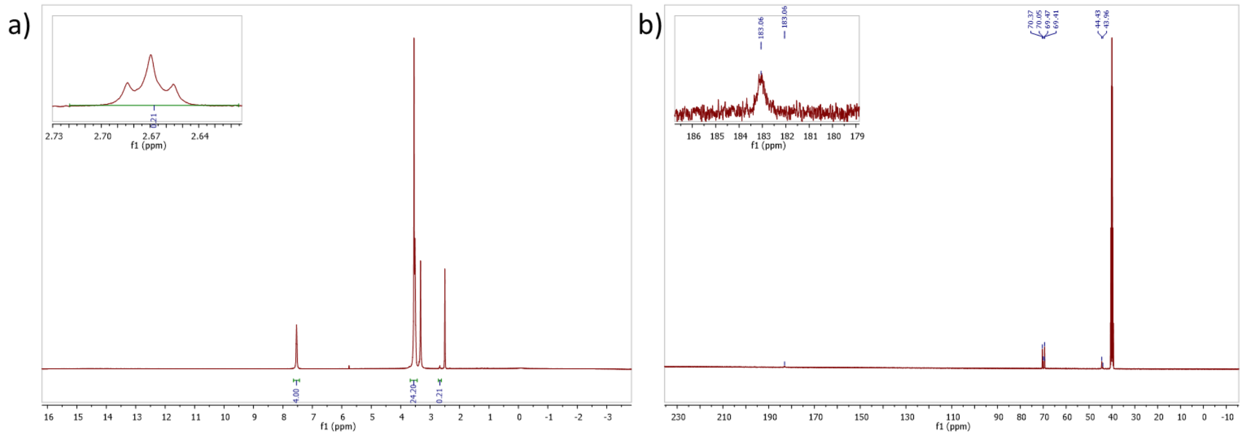

Figure S1 Nuclear Magnetic Resonance spectra of THA polymer: a) ^{1}H NMR, b) ^{13}C NMR.

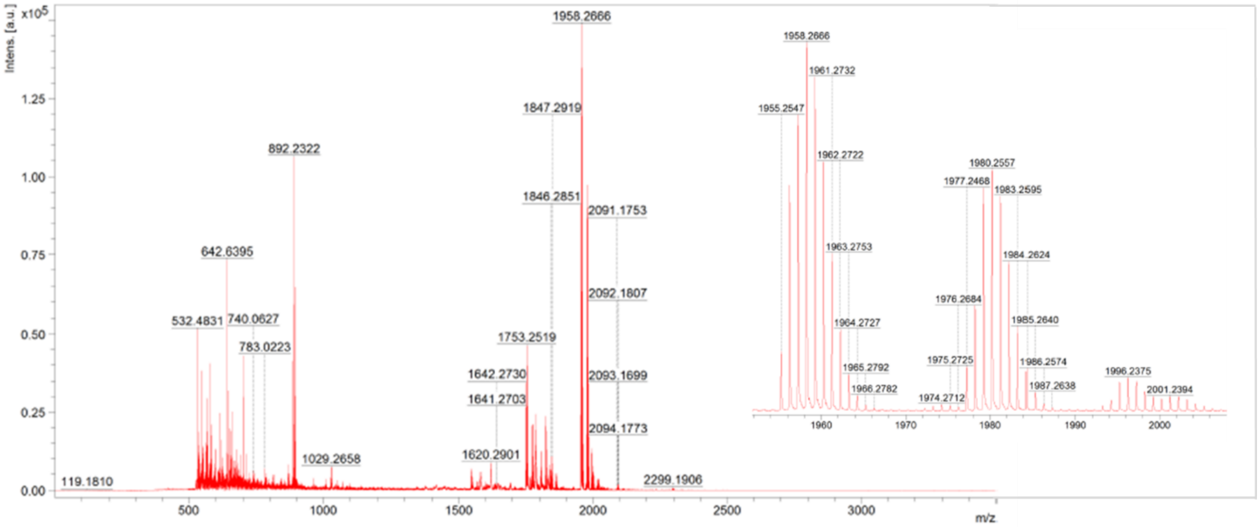

Figure S2 MALDI-TOF spectra of THA polymer.

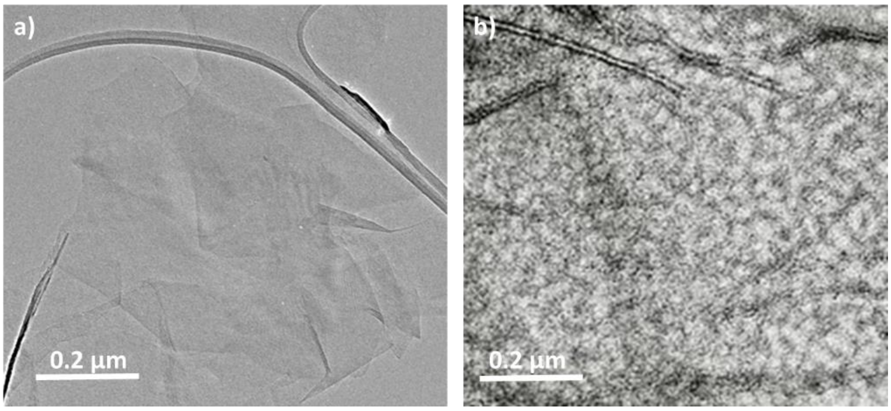

Figure S3 TEM pictures of (a) GO (b) GO-THA.

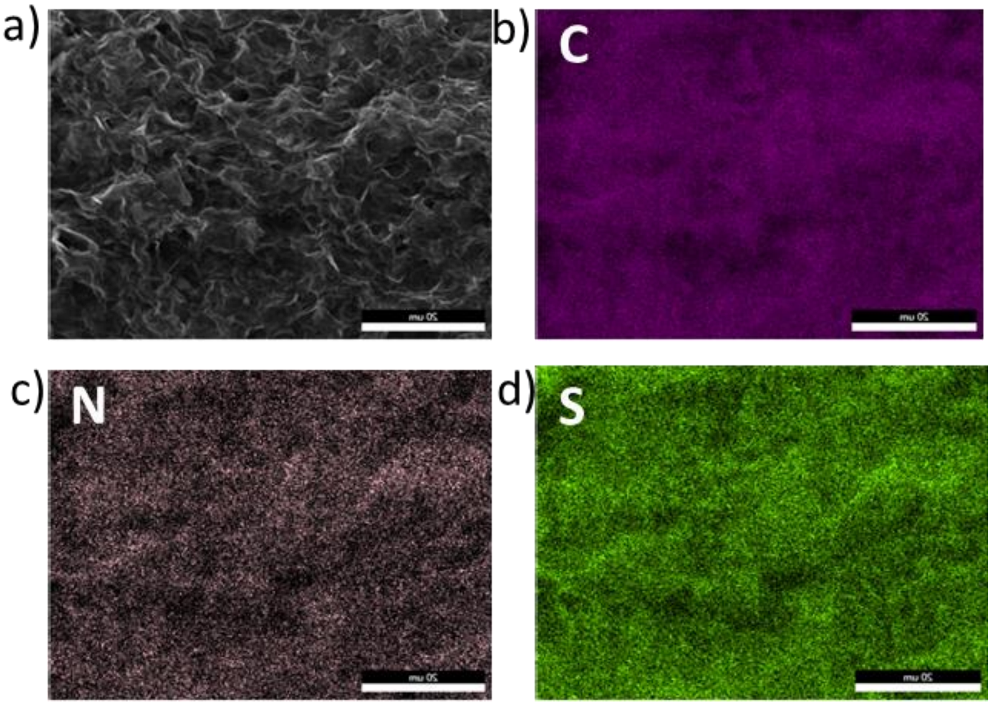

Figure S4 (a) SEM and (b-d) EDX element mapping spectra of GO-THA hybrid's (b) carbon, (c) nitrogen, and (d) sulfur.

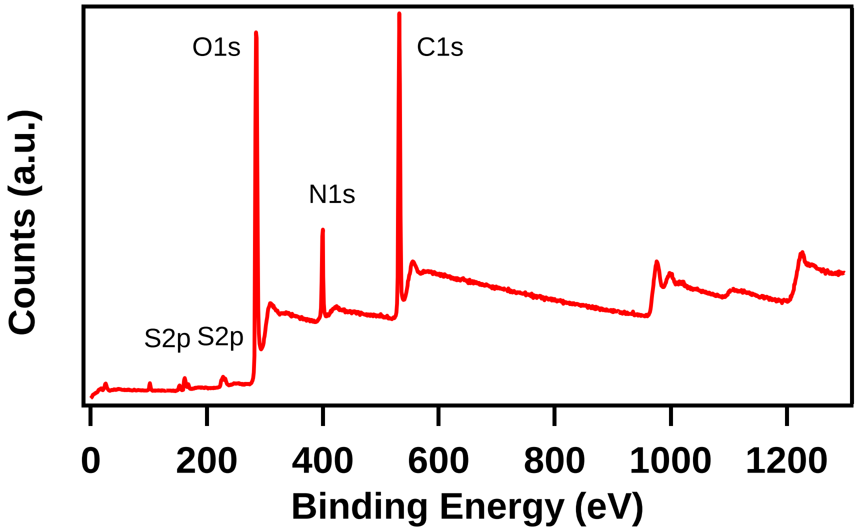

Figure S5 X-ray Photoelectron Spectroscopy survey spectra of GO-THA composite.

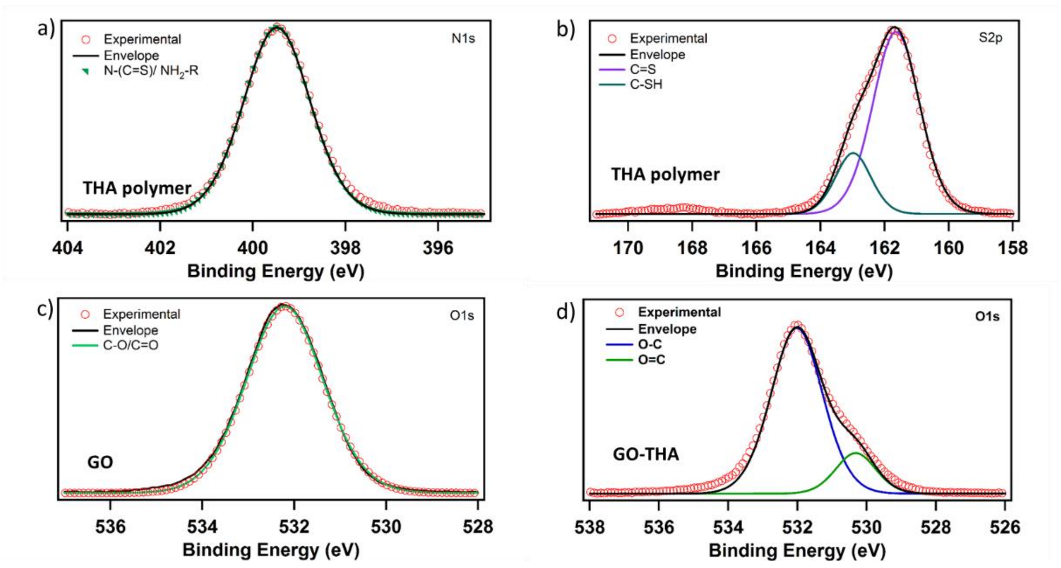

Figure S6 X-ray Photoelectron Spectroscopy of THA polymer: a) N1s, b) S2p, GO: c) O1s and GO-THA: d) O1s: d) O1s; GO-THA hybrid: e) O1s.

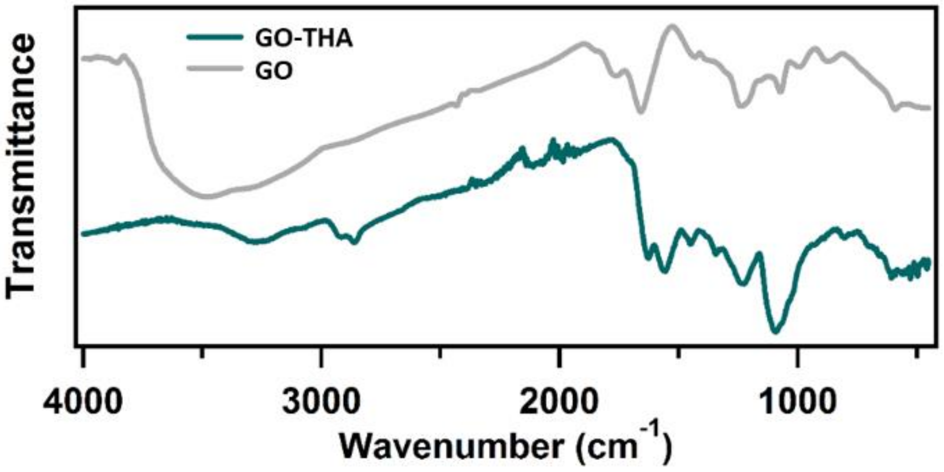

Figure S7 FT-IR spectra of GO (gray) and GO-THA hybrid (black).

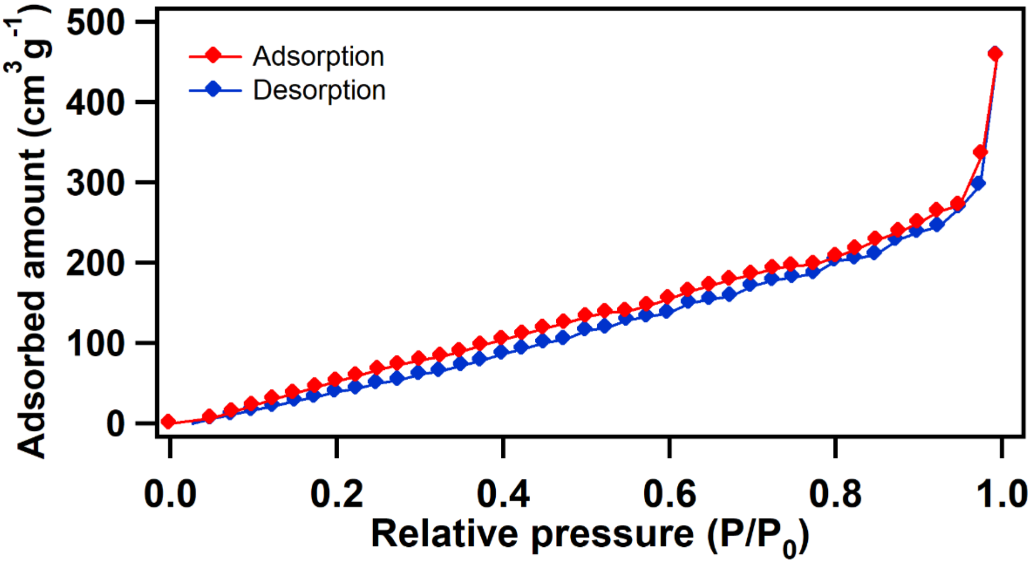

Figure S8 Adsorption-desorption N_2 isotherm of GO-THA hybrid.

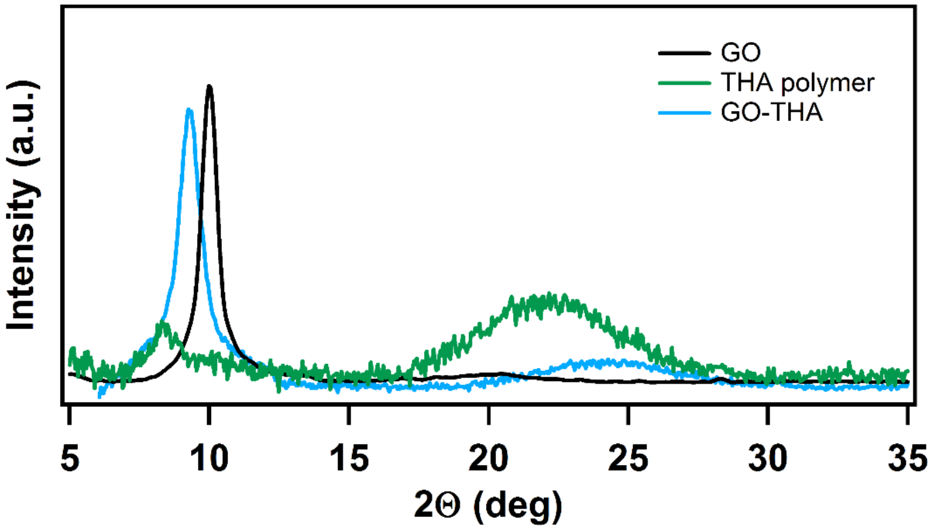

Figure S9 XRD patterns of GO, THA and GO-THA hybrid.

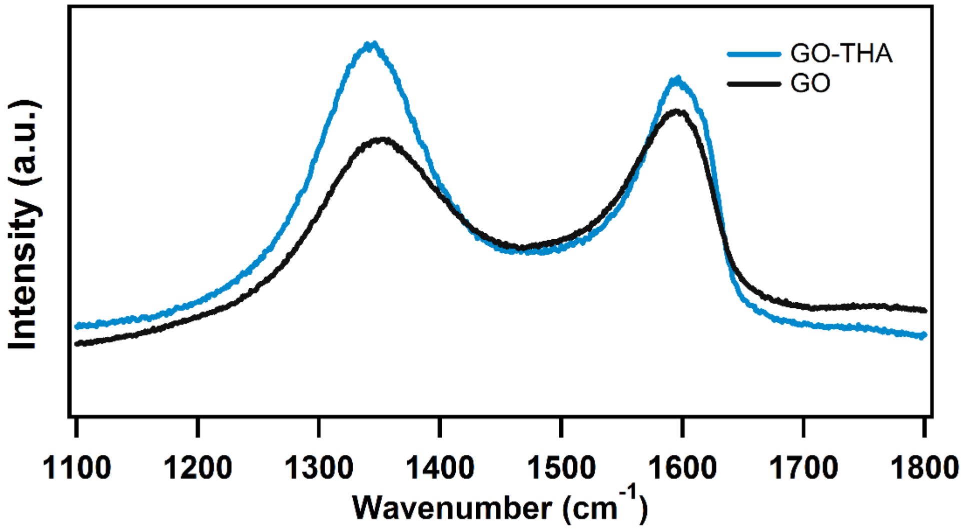

Figure S10 Raman spectra of GO and GO-THA hybrid.

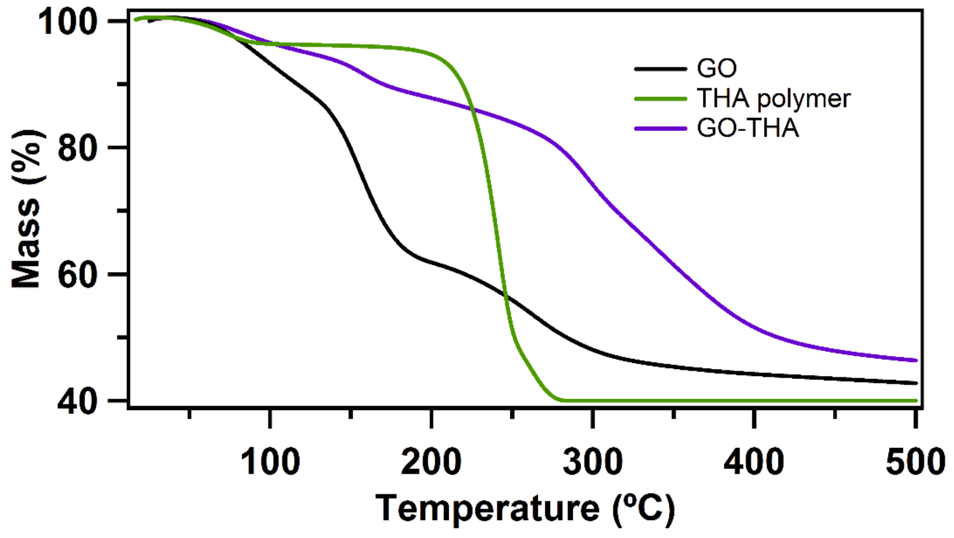

Figure S11 Thermogravimetric analysis of GO, THA and GO-THA hybrid.

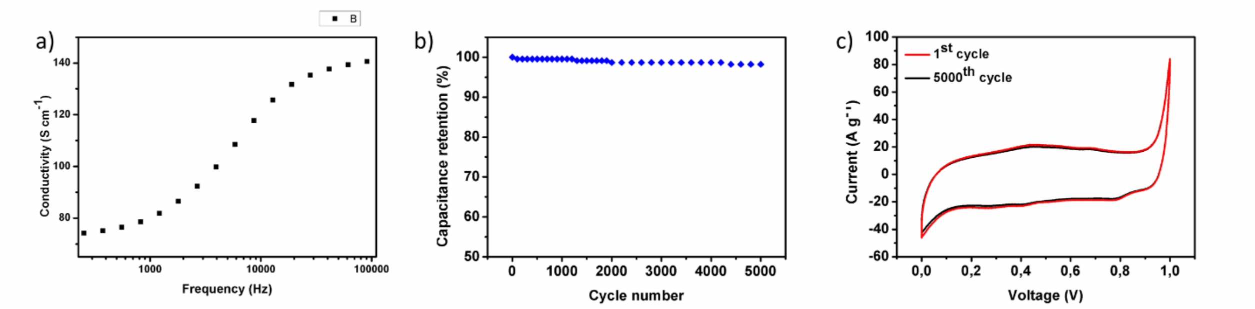

Figure S12 Electrochemical characteristics of GO-THA supercapacitor device in 1 M H_2SO_4: a) conductivity; b) stability of device over 5000 cycles; c) CV collected while 1st and 5000th cycle at 100 mV s^{-1}.

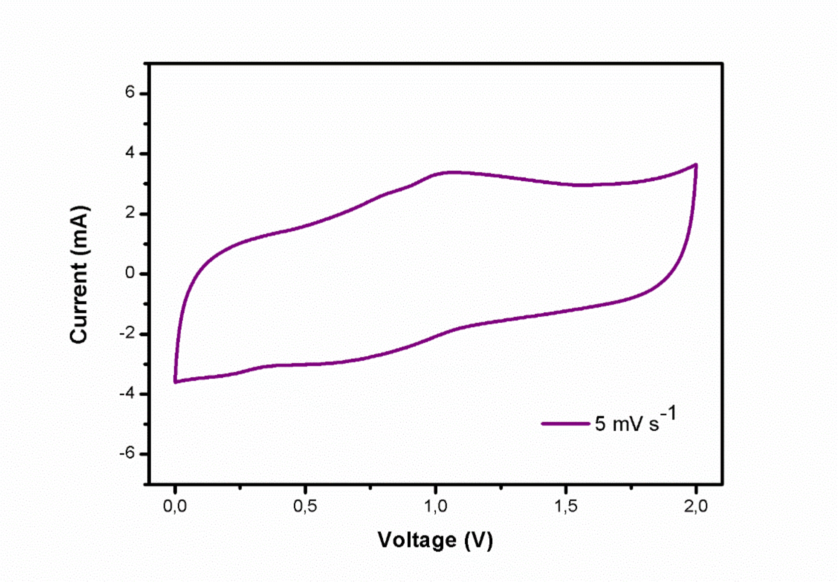

Figure S13 CV curve of GO-THA prepared device in $TEABF_4$ in ACN at 5 mV s^{-1}.

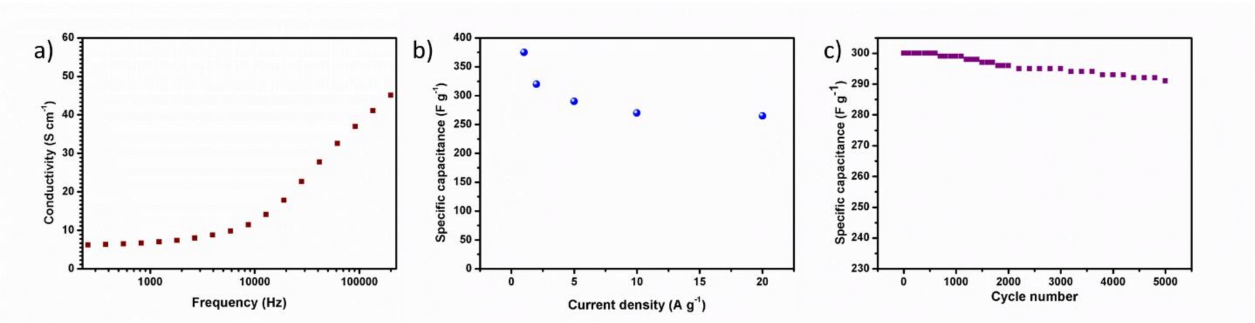

Figure S14 Electrochemical characteristics of GO-THA supercapacitor device in 1 M $TEABF_4$ in acetonitrile: a) conductivity; b) specific capacitance over different current density values; c) stability of the sample over 5000 cycles.

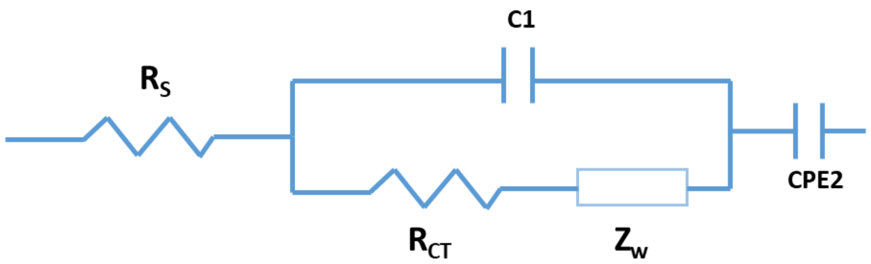

Figure S15 Proposed equivalent electrical circuit. Rs

Table S1 Chemical composition of GO-THA hybrid based on elemental analysis and XPS survey.

Table S2 BET surface area analysis of GO, POSS, rGO and rGO-POSS.

Analysis	%C	%N	%S
**Elemental analysis**	**55.07**	**9.17**	**4.02**
**XPS survey composition**	66.8	9.23	3.93

	BET Surface area (m^2g^{-1})	Pore volume ($cm^3\ g^{-1}$)	Average pore size (nm)
**GO**	108	0.15	5.6
**THA polymer**	62	0.013	2.0
**GO-THA**	371	0.0524	3.3

Table S3 Circuit parameters for the EIS measurements of GO-THA in 1M H_2SO_4.

Element	Parameter	Value	Estimated Error (%)
R_S	R	0.9	2.21

**R_{CT}**	R	6.0	3.54
**C1**	C	7.9 μF	7.12
**Z_W**	Y0	0.00596	12.5
**CPE2**	Y0	0.0036	6.84

Table S4 Circuit parameters for the EIS measurements of GO-THA in 1M $TEABF_4$ in ACN.

Element	Parameter	Value	Estimated Error (%)
**R_S**	**R**	4.6	2.17
**R_{CT}**	**R**	32	4.22
**C1**	**C**	12.9 μF	6.15
**Z_W**	**Y0**	0.00812	10.78
**CPE2**	**Y0**	0.00391	7.65

Table S5 The most important electrochemical parameters calculated for symmetric GO-THA device in 1M $TEABF_4$ in acetonitrile.

Current density ($A\ g^{-1}$)	Gravimetric capacitance ($F\ g^{-1}$)	Areal capacitance ($mF\ cm^{-2}$)	Volumetric capacitance ($F\ cm^{-3}$)	Energy density ($Wh\ kg^{-1}$)	Power density ($mWh\ cm^{-3}$)
1	371	556.5	85.6	94.4	24.1
2	324	486	74.8	90	41.5
5	289	433.5	66.7	80.3	92.6
10	275	412.5	63.5	76.4	176.3
20	260	390	60	72.2	333

Table S6 Comparison of most common characteristics of GO-polymer based electrodes.

Material	Specific capacitance	Scan rates/ Current density	Electrolyte	Energy density	Ref.
GO/polythiophene	201 $F\ g^{-1}$	10 mVs^{-1}	1M H_2SO_4	-	[1]
GO/PANI	320 $F\ g^{-1}$	0.1 $A\ g^{-1}$	2M H_2SO_4	-	[2]
GO	121.7 $F\ g^{-1}$<br>423 $F\ g^{-1}$	-<br>-	1M H_2SO_4<br>$C_4MIM\ BF_4$	3.08 $kW\ kg^{-1}$<br>11.6 $Wh\ kg^{-1}$	[3]
C-Sr-GO	260 $F\ g^{-1}$	1 $A\ g^{-1}$	6M KOH	36.3 $Wh\ kg^{-1}$	[4]
N-rGO	223.5 $F\ g^{-1}$	1 mVs^{-1}	6M KOH	-	[5]
NSG	176.5 $F\ g^{-1}$	1 $A\ g^{-1}$	6M KOH	-	[6]
GP	361.9 $F\ g^{-1}$	1 $A\ g^{-1}$	2M H_2SO_4	-	[7]
N/O-doped porous carbon	522 $F\ g^{-1}$ (3-electrode)	0.5 $A\ g^{-1}$	6M KOH	18.04 $Wh\ kg^{-1}$	[8]
Graphene/polyrrole	285 $F\ g^{-1}$	0.5 $A\ g^{-1}$	0.5M Na_2SO_4	-	[9]
N/S co-doped rGO	180.5 $F\ g^{-1}$	1 $A\ g^{-1}$	$EMIMBF_4$	33 $Wh\ kg^{-1}$	[10]
tris/rGO	119 $F\ g^{-1}$	10 mVs^{-1}	$BMIMBF_4$	51 $Wh\ kg^{-1}$	[11]

	131 F g^{-1}<br>189 F g^{-1}	2 mVs^{-1}<br>2 mVs^{-1}	$TEABF_4$/ACN<br>1M H_2SO_4	-<br>-	
pErGO	81 F g^{-1}	0.5 A g^{-1}	Polivinyl H_3PO_4 gel	11.25 Wh kg^{-1}	[12]
Sulfonated graphene PPy	310 F g^{-1}	0.3 A g^{-1}	1M KCl	4.3 Wh kg^{-1}	[13]
GO-THA	221 F g^{-1}<br>340 F g^{-1}	1 A g^{-1}<br>1 A g^{-1}	1M H_2SO_4<br>1M $TEABF_4$ ACN	27 Wh kg^{-1}<br>94.4 Wh kg^{-1}	This work

List of abbreviations: GO - graphene oxide; PANI- polyaniline; C-Sr-GO – crumpled sulfur-assisted reduced graphene oxide; N-rGO – nitrogen enriched reduced graphene oxide; NSG-nitrogen and sulfur co-doped graphene; GP- graphene-polyaniline, Tris/rGO- tris(2-aminoethyl) amine/ reduced graphene oxide, pErGO- growing porous electrochemically reduced graphene oxide, PPy- polypyrole.

References:

[1] N. A. Kumar, H. J. Choi, A. Bund, J. B. Baek, Y. T. Jeong, Journal of Materials Chemistry. **2012**, 22, 12268;
[2] K, Zhang, L. L. Zhang, X. S. Zhao, J. Wu, Chem. Mater. **2010**, 22,1392;
[3] M. P. Down, S. J. Rowley-Neale, G. C. Smith, C. E. Banks, ACS Appl. Energy Mater. **2018**, 1, 707;
[4] M. Hwang, H.-W. Kim, J.-U. Jin, H. Yoo, J. Yu, B.-C. Ku, N.-H. You, Int. J. Energy Res. **2021**, 45, 21209;
[5] L. Chen, X. Chen, Y. Q. Wen, B. X. Wang, Y. C. Wu, Z. T. Sheng, J. Nanosci. Nanotechnol. **2020**, 20, 4854;
[6] F. Pogacean,C. Varodi, M. Coros, I. Kacso, T. Radu, B. I. Cozar, V. Mirel, S. Pruneanu Biosensors, **2021**,11, 36;
[7] L. Jianhua, A. Junwei, Z. Yecheng, M. Yuxiao, L. Mengliu, Y. Mei, et al. ACS Appl. Mater. Interfaces, **2012**, 4, 2870;
[8] J. Lao, Y. Lu, S. Fang, F. Xu, L. Sun, et al. Nanomaterials, 2022, 12, 2186.
[9] P. Si, S. Ding, X. –W. Lou, D. –H. Kim, RSC Adv. **2011,** *1* (7), 1271.

[10] Y. Chen, L. Sun, Z. Liu, Y. Jiang, K. Zhuo, Mater. Chem. Phys. 2019, 238, 121932.

[11] B. Song, J. Zhao, M. Wang, J. Myllavey, Y. Zhu, et al. Nano Energy, 2017, 31, 183.

[12] T. Purkait, G. Singh, D. Kumar, M. Singh, R. S. Dey, Sci. Rep., 2018, 8, 640.

[13] X. Zuo, Y. Zhang, L. Si, B. Zhou, B. Zhao, L. Zhu, et al. J. Alloys Comp. 2016, 688, 140.
